# The Subtle Signaling Strength of Smells: A Masked Odor Enhances Interpersonal Trust

**DOI:** 10.3389/fpsyg.2019.01890

**Published:** 2019-08-20

**Authors:** Daan van Nieuwenburg, Jasper H. B. de Groot, Monique A. M. Smeets

**Affiliations:** ^1^Department of Psychology, Faculty of Social and Behavioral Sciences, Utrecht University, Utrecht, Netherlands; ^2^Department of Neurology, University of Pennsylvania, Philadelphia, PA, United States

**Keywords:** implicit, interpersonal trust, odor masking, olfaction, Trust Game, morphed faces, hexanal

## Abstract

Most everyday smells, from lavender to body odors, are complex odorant mixtures that “host” particular compounds that guide (social) behavior and motivation (biomarkers). A key element of social behavior is interpersonal trust, and building on previous research showing that (i) lavender odor can enhance trust, and that (ii) certain compounds in body odor can reduce stress in mice and humans (called “social buffering”), we examined whether a grassy-smelling compound found in both body odors and lavender, hexanal, would enhance interpersonal trust. Notably, we applied odor masking to explore whether trust could be influenced *subconsciously* by masked (i.e., undetectable) hexanal. In Study 1 (between-subjects), 90 females played a Trust Game while they either smelled hexanal (0.01% v/v), clove odor (eugenol: 10% v/v), or hexanal masked by clove odor (a mix of the former). As a sign of higher trust, participants gave more money to a trustee while exposed to masked hexanal (vs. the mask: eugenol). In Study 2 (within-subjects, double-blind), another sample of 35 females smelled the same three odors, while they rated the trustworthiness of a spectrum of faces that varied on trustworthiness. Controlling for subjective odor intensity and pleasantness and substantiating that masked hexanal could not be distinguished from the mask, faces were perceived as more trustworthy during exposure to masked hexanal (vs. the mask: eugenol). Whereas non-masked hexanal *also* increased face trustworthiness ratings, these effects disappeared after controlling for the odor’s subjective intensity and pleasantness. The combined results bring new evidence that trust can be enhanced implicitly via undetected smells.

## Introduction

The human sense of smell is better than generations of philosophers had thought (McGann, 2017). In subtle ways, odors can influence the way we perceive the world and act upon it, with approach and avoidance forming the most basic responses (Stevenson, 2010). Whereas “avoidance” is signaled by foul-smelling meat and most body odors including the smell of human fear (Pause, 2012; de Groot and Smeets, 2017; Parma et al., 2017), good smelling food and even the smell of a happy person could signal “approach” (Chen and Haviland-Jones, 2000; de Groot et al., 2015, 2018). As most smells are complex, consisting of hundreds of compounds, one of the main challenges is to identify key compounds that are instrumental in driving social behavior, with approach-related behavior being particularly overlooked in human olfaction research focusing mostly on malodor and negative emotions.

Chemical communication is ubiquitous in plants (e.g., Schilling et al., 2010) and the animal kingdom (e.g., Endres and Fendt, 2009). Within the bouquet of odorants that make up a smell, there arguably exist certain key compounds that have been preserved over the course of evolution and that consistently signal approach (mates with “good genes”; Havlicek and Roberts, 2009) or avoidance (predator odors; Endres and Fendt, 2009), even when embedded in a complex mixture. In humans, body odors from various sources have been found to trigger approach- and avoidance-related social responses, such as mothers’ breast milk odor causing crawling toward the odor source and suckling in babies (Schaal and Al Aïn, 2014), underarm odor from fearful individuals eliciting fear (Zhou and Chen, 2009; Pause, 2012; de Groot and Smeets, 2017; Parma et al., 2017), and female tears reducing sexual interest in males (Gelstein et al., 2011). As most research on human chemical communication has focused on social odors that elicit avoidance-related behaviors (fear, aggression, disgust), we attempted to balance the scale by examining whether certain odor compounds can facilitate appetitive behavior.

We searched the psychology literature for a target social communicative behavior with approach-related evolutionary significance where influence of odors has been previously demonstrated. We combined this with a search of the reviews of body odor volatiles for compounds that have emerged consistently over chemical analyses for such odors.

The approach-related behavior of interest was *interpersonal trust*, because trust is an important contributor to the maintenance, formation, and initiation of social relationships (Balliet and Van Lange, 2013) and inherent to social communication. Second, research has shown that trust-related behavior can be modulated by certain smells. Compared to control conditions (peppermint odor, no scent), the natural aroma of lavender increased the amount of money participants gave to a trustee (Sellaro et al., 2015b), a sign of trust. In another study, lavender odor was found to increased self-other integration (Sellaro et al., 2015a), which may help bridge perspectives between individuals and facilitate trust. Even outside of the lab, certain scents (e.g., orange odor) facilitated approach-related interpersonal behavior (e.g., dancing, drinking; Schifferstein et al., 2011). Since odor *masking* had not been applied in these studies, the query that remained open is whether trust can be enhanced *implicitly*, independently from the odors’ perceived pleasantness, by undetectable smells.

Masked odorants were found in another line of studies to reduce fear and stress, avoidance-related states inversely related to trust. Aside from studies showing *general* stress-reducing effects of *non-masked* smells, including orange odor (Lehrner et al., 2005) and lavender odor (Kritsidima et al., 2010), more recent studies in mice (Klein et al., 2015) and men (Endevelt-Shapira et al., 2018) have shown anxiolytic effects of an undetectable odorant present in body odor: hexadecanal. What underlined the *communicative* (social buffering) effect of hexadecanal is that typically developed humans showed attenuated fear-related startle responses to noise blasts during *masked* hexadecanal exposure, whereas this behavior was not observed in participants with autism spectrum disorder (Endevelt-Shapira et al., 2018). Rather than focusing on compounds that may attenuate avoidance-related states in already stressful situations, we examined whether particular compounds can facilitate *approach-related trust behavior* in a non-stressful situation, even when the odorant is masked and undetectable.

Departing from prior empirical studies showing the stress-reducing and trust-enhancing capacity of hexadecanal, orange odor, and lavender odor, we searched through the chemical analytical literature for “common denominator” compounds, with a particular focus on aldehydes – a class of chemical compounds generally associated with fruits, flowers (Schilling et al., 2010), and body odors (de Lacy Costello et al., 2014). From a list of 25 compounds most frequently isolated from headspace samples of human skin (Dormont et al., 2013), and based on a compendium of 1840 volatile organic compounds (VOCs) emanating from the human body (the volatolome; de Lacy Costello et al., 2014), we selected the aldehyde *hexanal* for the current research. When noticed, hexanal has the pleasant smell of freshly cut grass (Duke, 2015), but more often, hexanal remains “hidden” in mixtures that subserve some communication function. Indeed, gas chromatography-mass spectrometry (GC-MS) has revealed the presence of hexanal in body odor samples (Jha and Hayashi, 2017), human skin emanations (Pandey and Kim, 2011), where it may serve a social communication function, but also in lavender (ter Heide et al., 1970; Khan and Abourashed, 2010), a smell that was shown to enhance trust in humans (Sellaro et al., 2015a, b).

The main aim of this research was to test whether hexanal facilitated approach-related trust behavior in humans, and to dissect the subliminal contributions of *undetected* hexanal and the supraliminal effect of *consciously perceived* hexanal (HEX) to enhanced trust versus a commonly used mask (clove odor: eugenol). Buttressed by research showing that eugenol-masked compounds could still affect mood (androstadienone: Jacob and McClintock, 2000; Lundström et al., 2003), bias perception (androstadienone: Ye et al., 2019), and attenuate stress (hexadecanal: Endevelt-Shapira et al., 2018), we expected that eugenol-masked hexanal (HEX/EUG) would *implicitly* enhance trust relative to the mask odor (EUG). Furthermore, we expected that unmasked HEX would facilitate trust (vs. EUG) (Sellaro et al., 2015a, b), and that this supraliminal effect was driven by the odor’s explicit features, namely subjective odor pleasantness and odor intensity.

## Study 1: Materials and Methods

This research was conducted in accordance with the recommendations of Utrecht University’s Faculty Ethics Review Board, which approved our protocols (FETC17-033). All subjects gave written informed consent in accordance with the Declaration of Helsinki.

### Participants and Design

Ninety Utrecht University female undergraduates were recruited, based on power calculations for ANOVA (G^∗^Power; Faul et al., 2007), given *f* = 0.30, power = 0.80, α = 0.05, with effect size *f* taken from comparable research on lavender odor affecting interpersonal trust (Sellaro et al., 2015b). We recruited only women, because (i) women generally have a better sense of smell than men (Doty et al., 1984; Brand and Millot, 2001); (ii) women generally have stronger associations between smells and attributes of the physical/social environment than men (Kerr et al., 2005); and because (iii) our exploratory pilot study (sensitivity assessment in females and males) conducted before Study 1 (*N* = 48; *M*_*age*_ = 20.73 years; *SD* = 1.60) revealed a strong gender effect, *F*(1, 44) = 9.51, *P* = 0.004, *η_*p*_*^2^ = 0.18; *BF*_10_ = 3.93), with females showing enhanced trust responses (money given to a trustee) after (non-masked) hexanal exposure vs. odorless solvent (*n* = 30; *F*(1, 28) = 4.40, *P* = 0.045, *η_*p*_*^2^ = 0.14), but not males (*n* = 18); *F*(1, 16) = 1.60, *P* = 0.220. As smoking contributes to smell deficits and other sensory dysfunctions, smokers were also not included (Vennemann et al., 2008). All participants received €2 or course credit.

Participants were randomly assigned to *one* of three odor conditions (unmasked hexanal: HEX; hexanal masked by eugenol: HEX/EUG; mask odor: EUG).

### Materials and Measures

#### Odors

Unmasked hexanal (HEX) consisted of 0.01% hexanal (1 μL; CAS 66-25-1; 98% purity, Sigma-Aldrich) diluted in odorless propylene glycol (9.999 mL). Like in other research (e.g., Endevelt-Shapira et al., 2018), we used eugenol as control/mask odor (EUG): 10% eugenol (1 mL; CAS 97-53-0; 99% purity, Sigma-Aldrich) and 90% propylene glycol (9 mL). Masked hexanal (HEX/EUG) was a mixture (10 mL) of 10% eugenol (1 mL) and 0.01% hexanal (1 μL) diluted in 89.99% propylene glycol (8.999 mL). Odors were visually indistinguishable, and were contained in a transparent petri dish, with a coded label on the bottom only visible to the experimenter.

#### Trust Game

A customized, computerized version of the Trust Game (Berg et al., 1995) assessed the extent to which one person (trustor) trusts another person (trustee) (Camerer and Weigelt, 1988), as indicated by the amount of money transferred from trustor to trustee (Camerer, 2003; Capra, 2004; Sellaro et al., 2014). All participants were led to believe they would play one of two roles in a Trust Game with another “participant” (a confederate-experimenter who sat next door and who audibly knocked on the wall to indicate his/her presence). Unbeknownst to participants, they were all *trustors*, while the computer “played” the *trustee* (see section “Procedure,” for instructions). Participants were given €10, which they could keep or (partially) transfer to the trustee (marker of interpersonal trust). They were told that the transferred money would be tripled and that the trustee then decided if and how to share the tripled amount.

#### Implicit Affect

The Implicit Positive And Negative Affect Test (IPANAT) measured implicit (odor-induced) feelings through positive (happy, energetic, cheerful) and negative (helpless, tense, inhibited) “mood” ratings of non-existent words on 4-point Likert scales (Quirin et al., 2009).

### Procedure

Participants were individually seated based on odor condition (HEX, HEX/EUG, EUG) in one of three small (∼4 m^2^) cubicles (air circulation: 5 cycles/h). After completing 6 trials of the IPANAT (pre-test), the researcher opened the petri dish (containing the odor) that was held (∼3 cm below the nose) by an extension clamp, which was attached to a head-and-chin rest. Odors were renewed daily, and a lid prevented early odor dissipation and/or decay. During odor presentation, participants played the Trust Game, followed by a post-test IPANAT. For the Trust Game, participants received the following instructions: “You are going to play a game with another participant. One of you will be the PROPOSER, who decides how much money (€10) each player gets initially, after which the other person’s amount is tripled. The other person (RESPONDER) then decides how much of the tripled amount will be distributed between him/her and the proposer. The computer will randomly determine the roles at the start of the game. At the end of the game you will receive whatever amount is redistributed to you by the responder.” Then, the script indicated the processing of role assignment (2 s), after which the instructions continued: “In this game, you are the PROPOSER. You will be asked to distribute €10 between yourself and the responder. After you have pressed Enter, the responder will see how much you distributed to him/her. Please fill out how much money you want to give to the responder. You have 10 s to make your offer.” After another 10 s-waiting period, participants got the responder’s final response and were told the game was over. The whole game lasted less than 30 s. Next, olfactory function was established through correct identification of cinnamon, banana, and/or fish odor (Burghart Sniffin’ Sticks, Wedel, Germany; Lötsch et al., 2016), and 30 participants provided explicit hedonic ratings of odor pleasantness and intensity on 10-point Likert scales (1: “not at all …”; 10: “very …”). All participants were then debriefed and paid.

### Statistical Analysis

Eight out of 90 participants were excluded from data analysis, as they failed to correctly identify odors on the normosmia test. Since Trust Game data were not normally distributed, non-parametric tests were conducted (Kruskal–Wallis). Frequentist statistics were supplemented with Bayes Factors (*BF*_10_) estimating the likelihood of evidence for *H*_0_ (no difference) vs. *H*_1_.

## Study 1: Results

**FIGURE 1:**
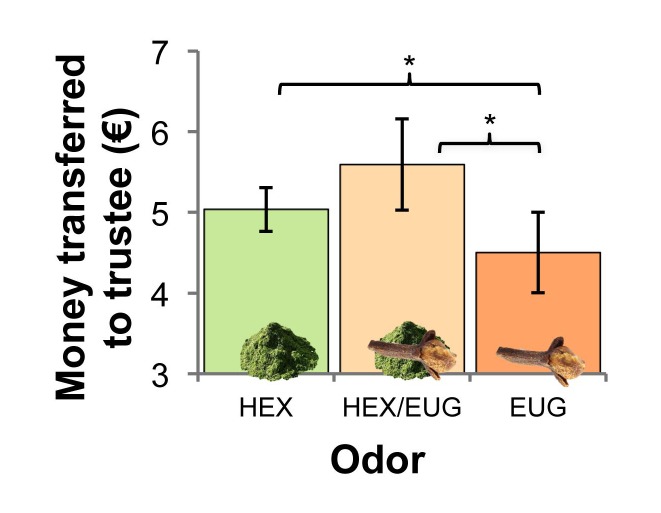
The (implicit) influence of “grass-like” hexanal (HEX) and hexanal masked by “clove smell” eugenol (HEX/EUG) vs. mask odor (EUG) on trust, indicated by money amount transferred to a trustee. Error bars ± 2 standard error of the mean (SEM). ^∗^*P* < 0.05.

We examined whether odors would impact interpersonal trust, indicated by how much money participants’ would transfer to a trustee in a Trust Game. A non-parametric Kruskal–Wallis test on Trust Game performance with between-subjects factor odor (hexanal masked by eugenol: HEX/EUG; hexanal: HEX; and mask odor: EUG) yielded a significant effect of odor, *H*(2) = 7.68, *P* = 0.021 (Figure 1). Follow-up planned comparisons revealed, as expected, that participants transferred more money to a trustee in the masked hexanal (HEX/EUG) condition (*N* = 27; *M* = 5.59 €; *SD* = 1.47) versus the mask (EUG) condition (*N* = 26; *M* = 4.50 €; *SD* = 1.27), *U* = 244.5, *P* = 0.017 (*BF*_10_ = 6.94; substantial evidence for *H*_1_ vs. *H*_0_). Participants also gave more money to a trustee in the unmasked hexanal (HEX) condition (*N* = 29; *M* = 5.03 €; *SD* = 0.73) versus EUG, *U* = 286.5, *P* = 0.018 (*BF*_10_ = 1.05; anecdotal evidence for *H*_1_ vs. *H*_0_), but no significant difference was found between HEX/EUG and HEX, *U* = 351, *P* = 0.382 (*BF*_10_ = 0.86, anecdotal evidence for *H*_0_ vs. *H*_1_).

### Control Measures

Next, we assessed whether odors induced changes in implicit positive and negative feelings pre- and post-odor presentation. A one-way ANOVA on these IPANAT scores revealed that odors neither induced changes in implicit positive affect [*F*(2, 79) = 1.49, *P* = 0.23], nor in implicit negative affect [*F*(2, 79) = 0.49, *P* = 0.62]. Hence, the effect of masked hexanal (HEX/EUG) on trust was not driven by changes in general implicit affective state.

Odors were renewed only on a daily basis and ∼3 participants were tested per day (*M*_*HEX/EUG*_ = 2.70; *SD*_*HEX/EUG*_ = 1.06; *M*_*HEX*_ = 2.90, *SD*_*HEX*_ = 1.10; *M*_*EUG*_ = 2.89, *SD*_*EUG*_ = 1.17); yet, Trust Game data were not impacted by testing order, HEX/EUG: *F* < 1 (*R*^2^ = 0.01); HEX: *F* < 1 (*R*^2^ = 0.02); EUG: *F*(1, 24) = 1.63, *P* = 0.213 (*R*^2^ = 0.06).

To check whether the odors’ explicit hedonic features could have boosted trust, we analyzed odor pleasantness and intensity ratings (*N* = 30). A Friedman test yielded significant differences in odor pleasantness, χ^2^(2) = 32.88, *P* < 0.001, and odor intensity, χ^2^(2) = 26.13, *P* < 0.001. A follow-up non-parametric test showed that HEX was perceived as significantly different from HEX/EUG (pleasant: *Z* = 4.119, *P* < 0.001; intense: *Z* = –4.04, *P* < 0.001), and from EUG (pleasant: *Z* = 4.58, *P* < 0.001; intense: *Z* = –4.42, *P* < 0.001), with HEX being more pleasant (*Mdn* = 8, *IQR* = 1) and less intense (*Mdn* = 5, *IQR* = 2), which could have boosted trust in that condition. However, since *masked* hexanal (HEX/EUG) and the mask (EUG) neither differed in perceived pleasantness (both: *Mdn* = 6, *IQR* = 1; *Z* = 1.31, *P* = 0.19), nor in perceived intensity (both: *Mdn* = 7, *IQR* = 2; *Z* = –1.75, *P* = 0.079), these explicit hedonic characteristics of smell could not have accounted for the observed trust increase that was specific to masked hexanal (HEX/EUG).

## Study 1: Discussion

Participants smelling masked hexanal (HEX/EUG) transferred more money to trustees than individuals smelling only the mask odor (EUG). This was the first demonstration of an undetectable, masked smell to enhance human trust. The effect of HEX/EUG could neither be accounted for by (changes in) implicit affect, nor by the odor’s subjective pleasantness and intensity. We sought to conceptually replicate this implicit odor-induced trust enhancement in another sample, using a face morph paradigm that allowed for collecting *repeated* trust ratings (vs. Trust Game’s “point estimate” of trust) in a double-blind within-subjects design to reduce inter-individual response variability thereby increasing statistical power.

## Study 2: Materials and Methods

### Participants

Another group of 35 female Utrecht University undergraduates (*M*_*age*_ = 21.26 years, age range: 18–32 years) participated in return for €6 or course credit. Sample size was based on a power analysis (G^∗^Power 3.1; *f*_*Study*__1_: 0.42; power: 0.80; α: 0.05). All participants had low scores (between 1 and 3) on the Modified Chemical Intolerance Index (Dalton, 1999), which asked if respondents felt sick after smelling cut flowers, pesticides, new carpeting, human body odors, et cetera (10 items; scores 1–5 “never” to “always”) to prevent oversensitive individuals from participating. No participant had to be denied because of high scores (≥3) on this index.

### Materials and Procedure

In this double-blind within-subjects experiment, both experimenter and participant remained unaware of the content of the odor bottles until debriefing, as bottles were coded by a researcher that was not involved in the experiment, while aluminum foil prevented potential visual discrimination of the odors.

Participants were individually presented with 10 ml of unmasked hexanal (HEX), masked hexanal (HEX/EUG), and the mask odor eugenol (EUG). The three odors were presented in a pre-determined counterbalanced order in a small glass bottle (100 mL), held by an adjustable clamp attached to a head-and-chin rest, ∼3 cm below the participant’s nose. The bottles were sealed with a lid prior to and directly following odor exposure to prevent stimulus dispersion and decay. Odors were renewed daily.

To test whether interpersonal trust would be subject to olfactory influence, participants smelled odors while completing a computer task, in which they rated the trustworthiness of a spectrum of faces varying on that dimension. The selected facial images were taken from a large pre-validated database of faces that were generated using FaceGen Modeller 3.2 (Todorov et al., 2013). In total, 18 face identities (Caucasian males) were used, with each identity varying in trustworthiness (5 levels: –2 SD, –1 SD, 0 SD, +1 SD, and +2 SD). We used only male faces, because FaceGen generates faces without scalp hair and male bald faces look more natural than female bald faces (Todorov et al., 2013). Each trial on the face judgment task consisted of a fixation cross (500 ms), followed by a brief (200 ms) face presentation (Todorov et al., 2009). Then, a visual analog scale (VAS) appeared, on which participants rated the trustworthiness of the preceding face from “not at all trustworthy” (0) to “very trustworthy” (100). There was a 1,500 ms intertrial interval. Each odor condition contained 30 trials (90 in total) and lasted no longer than 2 min.

Between odor conditions, participants did an unrelated filler task in a different cubicle (∼3 min), while the experimenter switched the odor bottle. After all three odors were presented, participants faced four psychophysical tests, to determine (i) normosmia (Lötsch et al., 2016), (ii) odor discrimination capacity (two-alternative forced-choice reminder task: 2-AFCR; (Van Hout et al., 2011), (iii) odor intensity (labeled magnitude scale: LMS; (Green et al., 1996), (iv) odor pleasantness (labeled hedonic scale: LHS; Lim et al., 2009), and (v) odor quality (whether odors smelled like clove or grass, to determine successful masking of HEX by EUG). Afterward, participants were debriefed and paid. No participant correctly guessed the masked odor hypothesis.

### Statistical Analysis

On the normosmia test, one participant could not identify cinnamon, banana, and fish; she was excluded (same criteria used in Study 1). The data analyst was blind to the odor conditions. In contrast to Study 1’s Trust Game, the morph task was susceptible to unusual response patterns and outliers: Two participants were excluded due to multivariate extreme scores (>3 SD ± group mean) on the face judgment task (Mahalanobis distance: *P* = 0.008), leaving *N* = 32. Remaining univariate outliers were identified, whenever applicable, using the median absolute deviation (MAD) (Leys et al., 2013). Like in previous research (de Groot et al., 2018; Kamiloglu et al., 2018), values that surpassed 2.5 MAD units ± the mean were altered to be one unit above the next extreme score that was not an outlier (Field, 2013). Odor discrimination data were analyzed with the SDT assistant (Hautus, 2012) and a one sample *t*-test comparing performance to chance (0.50). As odor intensity (but not pleasantness) ratings were not normally distributed, these data were log transformed.

## Study 2: Results

**FIGURE 2:**
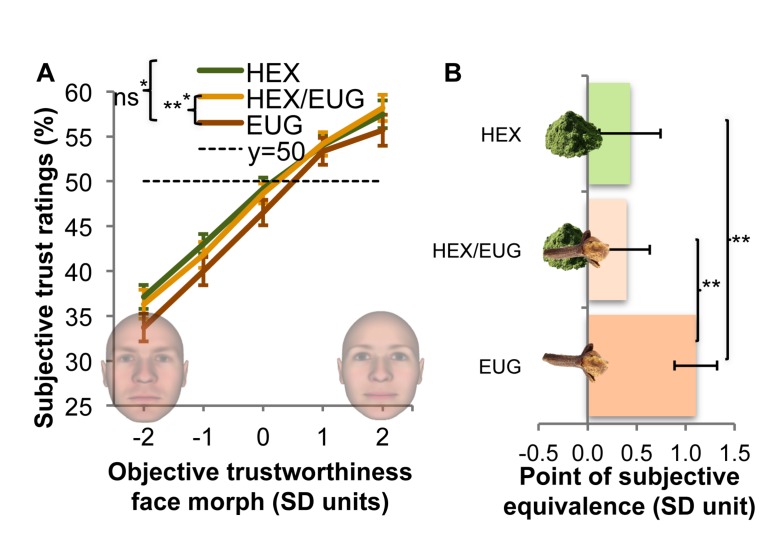
Effects of odor (hexanal: HEX, hexanal masked by eugenol: HEX/EUG; mask odor: EUG) on participants’ perceived trustworthiness of faces that objectively varied on trustworthiness (–2 to 2 SD). Main results are based on analyses controlling for subjective odor pleasantness and intensity. Error bars ± SEM. **(A)** Psychometric curves showing that overall trustworthiness ratings were higher for masked hexanal (vs. mask). The superscript result (^∗^) shows the same test without correction for pleasantness and intensity. **(B)** Masked and unmasked hexanal induced trust, evidenced by a significant shift (vs. mask) in the Point of Subjective Equivalence (PSE), the objective face at which 50% of subjective responses is “trustworthy” vs. “untrustworthy.” Lower values on the *x*-axis imply that faces objectively contained less trust. ^∗^*P* < 0.05; ^∗∗^*P* < 0.01.

In this double-blind within-subjects study, we used a face rating paradigm to test whether masked hexanal (HEX/EUG) would increase trust compared to the mask odor (EUG). To ensure the *implicitness* of this effect, participants’ explicit odor intensity and pleasantness ratings (see Control measures) were added as covariates^1^ to a 3 × 5 repeated measures ANCOVA on trustworthiness ratings, with odor condition (HEX, HEX/EUG, EUG) and face trustworthiness (SD units of trustworthiness: –2, –1, 0, +1, +2) as within-subjects factors. This analysis revealed, first of all, the expected main effect of odor, *F*(2, 54) = 4.76, *P* = 0.012, *η_*p*_*^2^ = 0.15 (Figure 2A), and face trustworthiness, *F*(4, 108) = 18.87, *P* < 0.001, *η_*p*_*^2^ = 0.41 (odor x face trustworthiness, *F* < 1). Regarding the main effect of odor, planned comparisons (adjusted for multiple testing: α/3 = 0.017) indeed revealed that participants identified faces as more trustworthy when exposed to HEX/EUG (*M* = 47.81%, *SD* = 6.17%) compared to EUG (*M* = 45.85%, *SD* = 6.25%), *F*(1, 27) = 15.03, *P* < 0.001, *η_*p*_*^2^ = 0.36 (*BF*_10_ = 1.23)^2^, whereas no significant differences were found between HEX and EUG (controlling for pleasantness and intensity), *F*(1, 27) = 2.97, *P* = 0.097 (*BF*_10_ = 1.26), and between HEX/EUG and HEX, *F*(1, 27) = 1.21, *P* = 0.281 (*BF*_10_ = 0.14; substantial evidence for *H*_0_ vs. *H*_1_).

Overall trustworthiness ratings were complemented by a specific analysis of a shift in participants’ Point of Subjective Equivalence (PSE), the objective point (in SD units) at which a face was equally likely to be deemed trustworthy or untrustworthy (Figure 2B). A single factor repeated measures ANCOVA revealed a significant effect of odor on PSE, *F*(2, 54) = 6.70, *P* = 0.003, *η_*p*_*^2^ = 0.20. Planned follow-up tests (α/3 = 0.017) showed that participants’ PSE significantly shifted to lower levels of objective face trustworthiness (indicating more trust) for HEX/EUG (SD units: *M* = 0.40; *SD* = 1.25) versus EUG (*M* = 1.10, *SD* = 1.80), *F*(1, 27) = 10.48, *P* = 0.003, *η_*p*_*^2^ = 0.28 (*BF*_10_: 3.03, substantial evidence for *H*_1_), and HEX (*M* = 0.43, *SD* = 1.24) vs. EUG, *F*(1, 27) = 8.020, *P* = 0.009 (*BF*_10_: 2.77; anecdotal evidence for *H*_1_). There were no significant differences between HEX/EUG and HEX, *F* < 1; to the contrary, Bayesian analysis yielded substantial evidence for *H*_0_, *BF*_10_: 0.14). Overall, these combined results replicate the trust-enhancing effects of hexanal, even when hexanal is undetectable when masked by eugenol.

Odors were renewed on a daily basis and ∼3 participants were tested per day (*M* = 2.92; *SD* = 1.51); yet, morph task performance could neither be predicted by daily testing order (across subjects), HEX/EUG: *F* < 1 (*R*^2^ = 0.02); HEX: *F* < 1 (*R*^2^ = 0.03); EUG: *F*(1, 31) = 1.06, *P* = 0.311 (*R*^2^ = 0.03), nor by *session* order (within-subjects), HEX/EUG: *F* < 1 (*R*^2^ = 0.00); HEX: *F* < 1 (*R*^2^ = 0.02); EUG: *F*(1, 31) = 1.16, *P* = 0.289 (*R*^2^ = 0.04). Prior exposure to unmasked odors (HEX or EUG) also did not bias subsequent morph task performance in the context of the masked odor (HEX/EUG), *F* < 1 (*R*^2^ = 0.04). Moreover, trustworthiness ratings on the morph task neither changed as a function of time (1st half of task, 2nd half of task), *F*(1, 31) = 1.89, *P* = 0.179, nor as a combined function of odor and time, *F* < 1.

### Control Measures

**FIGURE 3:**
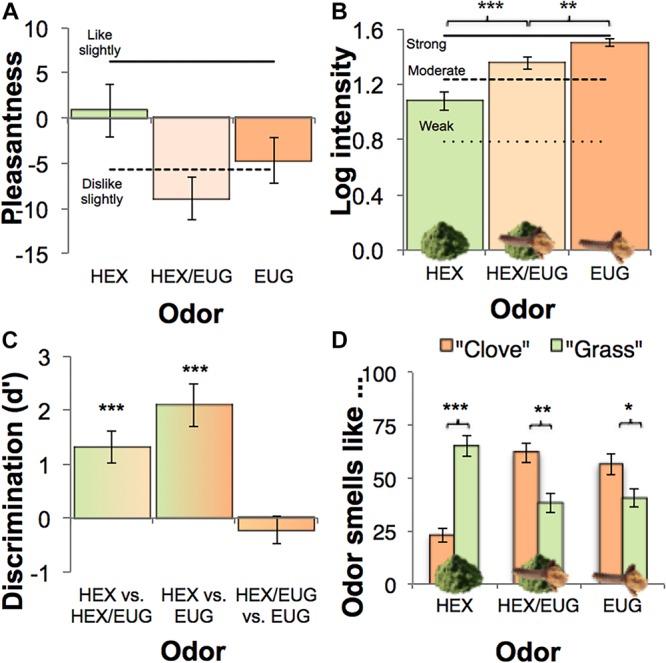
Control measures. Error bars ± SEM **(A,B,D)** or ±95% CI **(C)**. **(A)** Mean pleasantness on labeled hedonic scale. **(B)** Mean log transformed perceived intensity on labeled magnitude scale. **(C)** Odor discrimination performance, with values > 0 indicating significant discrimination. **(D)** Qualitative odor labels attached to the different odors. ^∗^*P* < 0.05; ^∗∗^*P* < 0.01; ^∗∗∗^*P* < 0.001.

An additional single factor repeated measures ANOVA was conducted to investigate whether the administered odors (HEX, HEX/EUG, EUG) were perceived differently in terms of pleasantness (labeled hedonic scale: LHS) and intensity (labeled magnitude scale: LMS). Whereas odors did not differ in perceived pleasantness, *F*(2, 62) = 1.82, *P* = 0.171 (Figure 3A), odor intensity did differ significantly across odors, *F*(2, 62) = 27.12, *P* < 0.001 (Figure 3B). Specifically, eugenol was rated as more intense (*M*_*log*_ = 1.50, *SD*_*log*_ = 0.16) than both hexanal (*M*_*log*_ = 1.08, *SD*_*log*_ = 0.36; *F*(1, 31) = 48.61, *P* < 0.001) and hexanal masked in eugenol (*M*_*log*_ = 1.36, *SD*_*log*_ = 0.26; *F*(1, 31) = 10.35, *P* = 0.003), whereas HEX/EUG was subjectively more intense than HEX, *F*(1, 31) = 17.15, *P* < 0.001. To control for these potential confounding factors, intensity and pleasantness difference scores were added as covariates to the aforementioned analyses of trustworthiness ratings.

The odor discrimination task (2-AFCR) results substantiated the claim that masked hexanal was indeed “masked” by eugenol (Figure 3C), as HEX/EUG could not be distinguished from EUG: *d*’ = –0.22, 95% CI [–0.48 – 0.04], *t*(31) = –1.07, *P* = 0.292. As expected, participants did correctly tell apart HEX from EUG (*d*’ = 2.10, 95% CI [1.69 – 2.50], *t*(31) = 14.73, *P* < 0.001), and HEX from HEX/EUG: *d*’ = 1.31, 95% CI [1.01 – 1.61], *t*(31) = 7.69, *P* < 0.001.

Furthermore, a 3 × 2 repeated measures ANOVA yielded a strong interaction between odor and the qualitative label (grass, clove) assigned to it, *F*(2, 62) = 29.89, *P* < 0.001, *η_*p*_*^2^ = 0.49: Hexanal smelled more “grass-like” than “clove-like”, *F*(1, 31) = 46.71, *P* < 0.001, whereas masked hexanal [*F*(1, 31) = 9.29, *P* = 0.005] and eugenol [*F*(1, 31) = 4.34, *P* = 0.046] smelled more like clove than like grass (Figure 3D).

## General Discussion

The main aim of this research was to examine whether a particular odor compound widely present in natural aromas and body odor, hexanal, would facilitate approach-related behavior, and to dissect its supraliminal and subliminal effect on trust. Based on prior research (Sellaro et al., 2015a, b; Endevelt-Shapira et al., 2018), we expected hexanal (when masked by eugenol, i.e., clove odor) to (i) *implicitly* enhance trust, compared to just the mask odor eugenol; and (ii), we tested whether detectable hexanal would *explicitly* facilitate trust compared to eugenol, an effect that could be ascribed to the odor’s subjective pleasantness and intensity. Whereas Study 1 employed a between-subjects design and recorded a “point estimate” of trust (endowing money to a trustee in a Trust Game), Study 2 measured continuous changes in the implicit perception of trust (judging morphed faces that varied in trustworthiness) in a double-blind within-subjects design. Two complementary experiments showed converging results, namely a moderately strong trust-enhancing effect for masked hexanal. Importantly, masked hexanal could *not* be discriminated from eugenol, and both smells were rated as qualitatively similar: “clove odor.” Furthermore, the trust-enhancing effects of masked hexanal were implicit; they could not be attributed to differences in subjectively perceived odor intensity and pleasantness.

Our findings show that hexanal is able to influence trust even outside of conscious awareness. As such, the study supplements existing research showing effects of undetectable smells on our perception and behavior (Degel and Köster, 1999; Li et al., 2007; Mujica-Parodi et al., 2009; Endevelt-Shapira et al., 2018). Using an olfactory affective priming paradigm, prior research (Li et al., 2007) has shown that subliminal odors (pleasant, unpleasant) can guide social likability judgments when odors were sufficiently *undetectable*. The reason is that participants who are in fact consciously aware of a stimulus (including a smell) may control (or reverse) their intuitive response in “top-down” fashion, called cognitive discounting (Kelley, 1973). When cognitive control cannot be exerted, such as when smells are undetectable or indistinguishable (like masked hexanal), the subcortical “high way” is followed from receptor to limbic regions (Gottfried, 2006), from where evolutionary ancient patterns of avoidance and approach behaviors are triggered. Here, we did not find evidence for cognitive discounting in the present study for unmasked, consciously detectable, hexanal; yet, when explicit ratings of odor pleasantness and intensity were *controlled for* (Study 2), we did find that faces were *not* rated as more trustworthy following exposure to unmasked hexanal versus eugenol. Indeed, without controlling for these subjective hedonic factors, we found anecdotal (Study 1) and substantial (Study 2) evidence for *no difference* between unmasked hexanal and masked hexanal on trust. That is, both unmasked and masked hexanal enhanced trust, but ostensibly via different processes, which is a novel finding future research could capitalize on (also see section “Limitations”).

Our findings dovetail with comparable research (Endevelt-Shapira et al., 2018) that showed a reduction in human avoidance responses (stress) following exposure to a longer-chain aldehyde (hexadecanal: C16) masked in eugenol (vs. eugenol). A calming, low-arousal trust state may play a role in this C16-induced “social buffering” effect, which has also been documented in mice (Klein et al., 2015). This social buffering process may rely on olfactory sensory neurons (OSNs) that express receptor OR37, which (i) belongs to a mammalian subfamily that is well conserved in mice and humans (Hoppe et al., 2003), and (ii) has high specificity for binding long-chain fatty aldehydes C15-C17 (Bautze et al., 2012). What may explain the role of hexadecanal (C16) in triggering subconscious olfactory responses is the fact that OR37 glomeruli target limbic brain regions (medial amygdala, hypothalamus) rather than typical olfactory cortical regions (Bader et al., 2012; Bear et al., 2016). At present, it cannot be ruled out that our short-chain aliphatic homolog hexanal (C6) activated OR37 as well; neither do we know the combinatorial code of C6 and C16 (Malnic et al., 1999), namely which ORs are (uniquely) activated by hexanal and hexadecanal. Given the discovery of a “family signature” of aldehydes in the olfactory bulb (OB), a certain degree of OR overlap is likely, with nuances in activity patterns along the spectrum of carbon chain length (Xu et al., 2003). A balance between receptor affinity and optimal volatility (short-chain hexanal is more volatile than long-chain hexadecanal) ostensibly determines a “best performing” compound to affect human behavior, but more research is needed to establish this.

### Limitations

Indeed, although this study brings new evidence in the form of a “proof of principle” that even a *masked odorant* can enhance trust, an important finding for the food and flavor industry, one clear limitation to our findings is the *specificity* of hexanal in enhancing trust when masked. At present, we cannot rule out that *other* aldehydes (or yet other compounds) would produce similar effect. The selection of hexanal was based on an extensive chemical database search for compounds frequenting human body odor (Dormont et al., 2013; de Lacy Costello et al., 2014), which was cross-matched with candidate compounds in natural aromas (lavender) that were empirically shown to reduce stress and increase trust (Kritsidima et al., 2010; Sellaro et al., 2015b). Using the present experimental approach, we could quickly chart the potential of a particular masked compound in affecting interpersonal trust (proof of principle); yet, for subsequent more complicated effectiveness assessments of aldehyde mixtures alongside their individual effects, data-driven machine-learning approaches may be preferred (Lötsch et al., 2018), which would draw on future databases of (body) odor samples and chemically analyzed components coupled to measures of social behavior.

Another limitation concerns the exclusive use of female participants, who were selected for this proof-of-principle study because of their greater sensitivity to olfactory influence versus males (affirmed by our pilot study), thus increasing the study’s potential for effectiveness. Within the female sample, various moderators could have affected the study’s outcome, including hormones fluctuating as a function of the menstrual cycle, and levels of empathy. Indeed, studies have shown that odor perception varies as a function of menstrual cycle phase (e.g., Doty et al., 1981; Pause et al., 1996), including perceived odor intensity and pleasantness – though not every tested odorant was affected (Hummel et al., 1991). In the present research, menstrual cycle phase was neither self-reported (a method that has been criticized: Blake et al., 2016), nor objectively tested (e.g., luteinizing hormone levels in urine); yet, this factor can arguably not explain the effects of masked hexanal on interpersonal trust, because subjective odor intensity and pleasantness had been controlled for in these analyses.

Another factor that could have influenced our results are individual differences in empathy. Whereas indeed, higher levels of empathy have been associated with better olfactory ability (Mahmut and Stevenson, 2016), our suggestion for future research is to focus on a more malleable factor that underlies empathy, olfactory ability, and trust: the neuropeptide oxytocin. Intranasal administration of oxytocin increased interpersonal trust in a trust game (Kosfeld et al., 2005) and perceived trustworthiness of faces (Theodoridou et al., 2009). Oxytocin has a double function by increasing empathy and attenuating stress responses (Rodrigues et al., 2009), which affects our brain (reduced amygdala activation) and body (reduced cortisol levels). In relation to smell, oxytocin diminished fear/stress responses following exposure to the smell of fear (Maier et al., 2019), while it improved detection of certain odors in schizophrenia patients (Woolley et al., 2015). Interesting questions related to the present research include participants’ *a priori* oxytocin levels (which could be explored for males as well, as oxytocin is gender-unspecific), how these levels would interact with particular smells like hexanal in promoting trust, and whether these interactions are similar for undetectable hexanal and perceivable hexanal.

## Conclusion

The present research brings new evidence to the literature (Sellaro et al., 2015b; Endevelt-Shapira et al., 2018) by demonstrating in two experiments (*N* > 100) the proof-of-principle that masked hexanal smell increased interpersonal trust (2 experiments; *N* > 100), indicated by more money given to a trustee (Study 1), and higher trustworthiness judgments of faces (Study 2). Psychometric tests and qualitative indicators corroborated that participants could not tell apart masked hexanal from the mask eugenol (clove smell), but despite that, only masked hexanal increased participants’ levels of trust (proof of principle). Our findings highlight the subconscious impact of smells, with human olfaction – despite being historically derogated prior to careful empirical consideration – proving more powerful than initially thought (McGann, 2017).

## Data Availability

The datasets generated for this study are available on the Open Science Framework: osf.io/48rbx/.

## Ethics Statement

This study was carried out in accordance with the recommendations and guidelines of Utrecht University’s Faculty Ethics Review Board with written informed consent from all subjects. All subjects gave written informed consent in accordance with the Declaration of Helsinki. The protocol was approved by Utrecht University’s Faculty Ethics Review Board (FETC17-033).

## Author Contributions

JdG designed both experiments. MS co-designed Study 2. DvN programmed and conducted the experiments. DvN and JdG analyzed the data. DvN wrote the first draft with input from JdG and MS. JdG wrote subsequent versions of the manuscript in consultation with DvN and MS.

## Conflict of Interest Statement

The authors declare that the research was conducted in the absence of any commercial or financial relationships that could be construed as a potential conflict of interest.

## Footnotes

1 Analyses without the covariates intensity and pleasantness revealed no difference between masked (unperceivable) and unmasked (perceivable) hexanal on trust ratings, *F* < 1, whereas there were significant differences between masked hexanal (HEX/EUG) and eugenol, *F*(1, 31) = 4.84, *P* = 0.034, *η_*p*_*^2^ = 0.14, and between unmasked hexanal (HEX) and eugenol. *F*(1, 31) = 4.89, *P* = 0.035, *η_*p*_*^2^ = 0.14).

2 Odor intensity and pleasantness were not controlled for when calculating Bayes factor.
